# The longitudinal effect of perceived person-environment fit on academic trajectories and the mediating role of self-concepts and expectations of success

**DOI:** 10.1007/s10212-025-01058-x

**Published:** 2026-01-19

**Authors:** Lukas Ramseier, Markus P. Neuenschwander

**Affiliations:** Center for Learning and Socialization, University of Applied Sciences and Arts Northwestern Switzerland, Bahnhofstrasse 6, CH-5210 Windisch, Switzerland

**Keywords:** Person-environment fit, Self-concepts, Motivation, Educational trajectories

## Abstract

In Switzerland, a goal during lower secondary school is to secure a qualifying upper secondary education, either vocational education and training or general education. By combining person–environment fit theory and expectancy–value theory, it is assumed that students’ perceived person–environment fit in primary school is associated with whether they enter a non-qualifying upper secondary education, mediated by self-concepts and expectations of success in mathematics and German. To test this mediation, a longitudinal structural equation model (SEM) was constructed, using data from 388 Swiss students at three measurement points. The results show that students’ perceived person-environment fit in sixth grade indirectly and negatively predicted them entering a non-qualifying upper secondary education after completing ninth grade. The effect was mediated by self-concepts in mathematics and German as well as expectations of success in mathematics. The assumed effect of expectations of success on a non-qualifying upper secondary education was not significant, indicating that there are domain-specific mechanisms underlying the effects of person-environment fit and motivational beliefs on educational trajectories. Implications for theoretical conceptualization and educational practice are discussed.

## Introduction

Preventing educational dropout is a key objective of educational systems internationally, as completion of upper secondary school is seen as a necessity across many OECD countries (Lyche, 2010; Wilson et al., 2011). In Switzerland, to reduce the risk for unemployment and social exclusion, the Swiss Conference of Cantonal Ministers of Education (EDK) has formulated the goal that 95% of all adolescents should achieve an upper secondary qualification (SCCRE, 2023). Delays in entering a qualifying upper secondary education significantly reduce the likelihood of eventually completing such a qualification at all (Meyer, 2018). Unfortunately, in Switzerland, nearly 15% of all adolescents are unable to find such a program by the end of lower secondary school and enter a non-qualifying upper secondary education (SCCRE, 2023). It is therefore critical to identify factors that increase the probability of this outcome.

A promising approach to explain students’ choices and achievements is the theory of person-environment fit (P-E-F; Eccles et al., 1993; Hunt, 1975). It posits that an optimal match between an individual’s characteristics and their learning environment is an important aspect of optimal developmental conditions and leads to positive academic outcomes, such as qualifying educational pathways and achievements (e.g., academic persistence, work satisfaction, or educational attainment; de Vries et al., 2024; Rauvola et al., 2020; Suhlmann et al., 2018).

The present study aims to integrate P-E-F theory with expectancy-value theory (Eccles & Wigfield, 2002), which suggests that educational choices and achievements are directly predicted by motivational beliefs, such as self-concepts and expectations of success. It can be assumed that these beliefs mediate the positive effect of a high P-E-F on students’ educational choices and achievements.

Perceived P-E-F has been found to be associated with various motivational beliefs (Ramseier & Neuenschwander, 2024a; Ramseier & Neuenschwander, 2024b; Zimmer-Gembeck et al., 2006), including self-concepts and expectations of success (Gerber-Schenk et al., 2010). However, so far, no longitudinal study has tested whether students’ self-concepts and expectations of success mediate the effect that perceived P-E-F has on their educational trajectories. Furthermore, perceived P-E-F has only rarely been combined with expectancy-value theory and explicitly analyzed as a distinct construct.

By supplementing expectancy-value theory with P-E-F theory, the present study examines whether students’ perceptions of their school environment (P-E-F) in primary school are prospectively associated with their motivational beliefs (self-concepts and expectations of success) in lower secondary school, and whether these beliefs, in turn, are negatively associated with their likelihood of entering a non-qualifying upper secondary education. Combining the two theoretical frameworks is a promising approach to a more nuanced understanding of the interactions between environmental learning conditions, students’ beliefs, and their educational achievements and choices.

### Upper secondary education in Switzerland

Upper secondary education in Switzerland mainly consists of two tracks that most adolescents take advantage of: general education and vocational education and training (VET; SCCRE, 2023). General education courses prepare students for tertiary level training courses (mainly universities). In VET, on the other hand, adolescents start an apprenticeship and learn a profession. It is mostly completed in training companies with additional school lessons (a dual system). Around two thirds of adolescents complete VET after lower secondary school, and around one quarter completes general education (SCCRE, 2023). Both tracks enjoy a good reputation in Switzerland and are considered qualifying training courses that are completed with a diploma. The completion rate of a qualifying upper secondary education is 91.4% (SCCRE, 2023).

However, not all adolescents manage to enter one of these two tracks right after lower secondary school. They instead have to choose a non-qualifying interim solution, e.g., an internship. These interim solutions are not part of the intended educational trajectory and mainly serve as temporary steps toward entering a qualifying track, but are not qualifying themselves. However, with every year in which an adolescent is unable to find a qualifying upper secondary education, the risk of not being able to find one at all increases (Meyer, 2018). It is therefore a goal to secure a qualifying upper secondary education at the end of lower secondary education. There is, however, an increasing trend towards interim solutions: in 2000, 12.4% of all adolescents in their first year of upper secondary education were in some sort of interim solution. In 2018, this number had increased to 14.9% (SCCRE, 2023). Identifying factors associated with adolescents’ choice of a non-qualifying upper secondary education is therefore crucial.

### Perceived person-environment fit

P-E-F refers to the extent to which the current learning environment matches individual characteristics (Eccles et al., 1993), specifically abilities, needs, and interests (Brown & Lent, 2005; Etzel & Nagy, 2015). According to P-E-F theory (Eccles & Roeser, 2009, 2011; Eccles et al., 1993; Hunt, 1975), the extent to which a learning environment fits students’ abilities, needs, and interests predicts academic outcomes in subsequent school years, such as learning engagement and even withdrawal from school (Finn, 2006). Empirical findings support this assumption and suggest that P-E-F predicts longer-term decisions, including those related to upper secondary education and school dropout (Findeisen et al., 2023; Neuenschwander et al., 2017; Roberts & Robins, 2011).

A high fit between the student and their educational environment creates favorable conditions for positive development, resulting in educational achievement as well as academic aspiration and attainment (Neuenschwander, 2017; Kühner et al., 2024). According to research, perceived P-E-F is especially relevant in this regard. Perceived P-E-F refers to the P-E-F directly perceived by the individual and not assessed by objective criteria. Nägele et al. (2017) showed that adolescents’ perceived P-E-F was a stronger predictor of their job satisfaction and their intention to complete further training than an objectively measured P-E-F. Perceived P-E-F can therefore be seen as a valid predictor of educational choices and will be the focus of the present study.

According to P-E-F theory, a high perceived P-E-F at school helps students keep psychologically engaged, which results in higher levels of educational attainment (Eccles & Roeser, 2009; Steinhoff & Buchmann, 2017). Previous studies support these assumptions and show that individuals who perceive their learning environment as fitting their abilities, needs, and interests report higher levels of engagement (Aldridge et al., 2024; Kühner et al., 2024; Zimmer-Gembeck et al., 2006) or goal orientation (Ramseier & Neuenschwander, 2024a; Ramseier & Neuenschwander, 2024b). This is also reflected in educational trajectories at the end of lower secondary school: perceived P-E-F has been found to be a predictor for educational choices and persistence at the upper secondary or higher education level (de Vries et al., 2024; Le et al., 2014).

It can be assumed that students’ perceived P-E-F is not only related to their current beliefs and achievements, but also shows longitudinal associations: An individual’s mental representation of their environment can be transferred from one context to another (Hofmann & Venetz, 2017). Experiences made in a certain learning environment are crucial for motivational beliefs shaped in subsequent learning environments (Hofmann & Venetz, 2017; Waters et al., 2014). Experiencing the learning environment in primary school as fitting, therefore, helps students adapt to the new context in lower secondary school.

Based on these theoretical assumptions and empirical findings, it can be assumed that students’ perceived P-E-F in primary school (sixth grade) favors their motivational beliefs in lower secondary school and thus has a long-term, sustainable effect on their educational path, particularly in preventing them from entering non-qualifying upper secondary education after lower secondary school.

### Motivational beliefs: expectations of success and self-concepts

Motivation refers to an individual’s beliefs that determine the tasks they choose and the amount of effort they invest (Lazarides & Raufelder, 2017; Wentzel & Wigfield, 2009). Motivational beliefs are a crucial factor concerning which upper secondary education (within the existing options) is chosen at the end of lower secondary education. According to expectancy–value theory (Eccles & Wigfield, 2002), expectations of success and task values are key beliefs in predicting these choices.

Values refer to the interest, importance, usefulness, and costs that are attributed to a particular task (Eccles & Wigfield, 2002). They are mainly predictive for the content orientation of the chosen education (the “horizontal” dimension; Neuenschwander, 2017; Sander & Kriesi, 2021). Expectations of success are defined as an individual’s belief in how well a particular task can be accomplished, either in the immediate or distant future (Fasbender, 2018; Feather, 1982). They are crucial when it comes to the performance level of a chosen education (the “vertical” dimension; Neuenschwander, 2017; Sander & Kriesi, 2021). Since the present study aims to explain the lack of a qualifying upper secondary education (a vertical educational choice), the focus will be on expectations of success exclusively.

Expectations of success describe a construct that is closely related to Bandura’s (1997) concept of self-efficacy, insofar as both self-efficacy and expectations of success refer to a person’s expectations concerning success in future tasks (Eccles & Wigfield, 2002; Schoenherr, 2024;

Schweder & Raufelder, 2022). An important aspect of expectations of success is their domain specificity, meaning that they do not necessarily equally apply to all areas, but can differ between subjects (Eccles, 1983). For example, a student might have high expectations in mathematics, but not in German (and vice-versa). This is reflected in the results of several studies that did not find a correlation between students’ expectations of success or related constructs like self-efficacy in different subjects (Peklaj et al., 2006; Smith & Fouad, 1999).

It can be assumed that expectations of success in mathematics and German are particularly relevant concerning the chosen upper secondary education, since these two subjects are the most crucial for the transition to upper secondary level in Switzerland (Haeberlin et al., 2005). In the German-speaking part of Switzerland, mathematics and German serve as key selection criteria to lower and upper secondary school tracks across all cantons (EDK, 2022; EDK, 2022; SCCRE, 2023), making them highly relevant for educational transitions.

According to Eccles and Wigfield (2002), expectations of success are directly predicted by self-concepts. Self-concept refers to the self-evaluation of one’s abilities in a certain domain (Marsh & Martin, 2011; Moschner & Dickhäuser, 2006). Early research on self-concepts assumed it as a global construct (Coopersmith, 1967); however, modern consensus sees it as domain-specific (Laskowski, 2000), akin to expectations of success. Findings show that students’ self-concepts are lower in areas in which they have learning difficulties, but that this low self-concept does not necessarily transfer to other subjects (Schuchardt et al., 2015). This aligns with current theoretical frameworks, which posit that self-concept develops within specific academic domains rather than as a generalizable trait (Rost & Feng, 2024). Consequently, it can be assumed that self-concept in distinct subjects, like mathematics and German, is an independent construct rather than correlated (Schuchardt et al., 2015; Smith & Fouad, 1999).

Expectations of success and self-concepts are related constructs, and the terms are often used interchangeably (jingle fallacy; Jansen et al., 2015; Marsh et al., 2019). There are, however, certain crucial distinctions: While self-concepts refer to perceived abilities in specific domains, based on previous achievements, expectations of success are future-oriented evaluations of the capability to succeed in a given area or at a task (Bong & Clark, 1999; Bong & Skaalvik, 2003). Both self-concepts and expectations of success can—among other beliefs—be summed up under the banner of “motivational beliefs,” but while expectations of success can be described as “task-related,” self-concepts are more “person-related” (Han, 2017; Midgley et al., 1989; Schiefele, 2009).

This distinction is also present in expectancy-value theory: It posits that self-concepts of one’s abilities directly influence expectations of success, which in turn predict educational choices (Eccles, 1983; Eccles & Wigfield, 2002). If an individual has a high self-concept in a particular subject or area—for example, due to positive learning experiences, previous successes, or constructive feedback from teachers—(i.e., they have a positive idea of their abilities), they will also have positive expectations of success in the respective subject or area (i.e., they expect to be able to master tasks). Empirical research shows that self-concepts are associated with expectations of success (Dickhäuser & Reinhard, 2006; Midkiff et al., 1986; Urdan & Argueta-Vogel, 2018).

It can therefore be assumed that a pronounced self-concept in relevant subjects, such as German and mathematics, during lower secondary school indirectly reduces the likelihood of students entering a non-qualifying upper secondary education, with the effects being mediated by expectations of success in the respective subjects. This is particularly relevant in the Swiss context, where these two subjects are central to selection decisions and play a crucial role in determining students’ educational trajectories (EDK, 2022; EDK, 2022; SCCRE, 2023, 2023).

According to expectancy-value theory, students’ self-concepts and expectations of success in educational settings are informed by their perceived engagement with their learning environment (Eccles & Wigfield, 2002). While expectancy-value theory does not explicitly name P-E-F as a variable, it highlights the importance of students’ perceptions of their environment and their relationship with that environment. Within this framework, perceived P-E-F can be understood as a specific perception of this relationship—namely, the perceived alignment between one’s own characteristics and the educational environment—that informs students’ motivational beliefs about themselves as learners. The integration of P-E-F theory—which highlights the crucial role of perceived alignment between an individual’s abilities, needs, and interests with their environment in fostering motivational beliefs (Eccles & Roeser, 2009, 2011; Eccles et al., 1993)—provides a more nuanced rationale for this process. When students perceive a high degree of fit between their personal characteristics and the learning environment, they are more likely to feel competent and in control, conditions known to foster positive academic self-concepts (Marsh & Craven, 2006; Tonkin & Watt, 2003). Conversely, environments that are perceived as mismatched may signal to students that they lack the ability to succeed, potentially undermining their self-concept.

Empirical research confirms these assumptions, demonstrating that students who perceive their learning environment as fitting their characteristics are more likely to be motivated and experience higher engagement (Ramseier & Neuenschwander, 2024a; Ramseier & Neuenschwander, 2024b; Zimmer-Gembeck et al., 2006). Specifically, it is assumed that when students perceive a high P-E-F, they are more likely to develop a positive self-concept in specific academic domains. The reason for this is that an environment that is not perceived to match one’s needs, abilities, and interests leads to a feeling of being overwhelmed by academic demands, which can manifest as a lower self-concept in individual domains (Tonkin & Watt, 2003). Empirical research has shown that perceived P-E-F predicts individuals’ domain-specific self-concepts (Gerber-Schenk et al., 2010). By incorporating P-E-F theory into the expectancy-value framework, the present study posits that perceived P-E-F informs students of their academic abilities and is associated with domain-specific self-concepts and expectations of success. Self-concepts and expectations of success, in turn, mediate the favorable effects perceived P-E-F has on educational choices and achievements. Thus, incorporating P-E-F into the expectancy-value model enhances our understanding of how students’ perceptions of fit within their learning environment translate into decision-making in academic settings.

### The present study

Studies on students’ perceived P-E-F at school and its direct and indirect effects on motivational beliefs and subsequent non-qualifying educational choices are still rather scarce. The present study analyzes whether students’ perceived P-E-F shows a longitudinal effect on their self-concepts and expectations of success in mathematics and German and whether these beliefs, in turn, predict them not obtaining a qualifying upper secondary education.

This assumed mediation of the effects of perceived P-E-F on the upper secondary educational choice by self-concepts and expectations of success will be tested in a longitudinal design, incorporating three measurement points, namely sixth grade (final year of primary school; *t*_1_), seventh grade (first year of lower secondary school; *t*_2_), and ninth grade (final year of lower secondary school; *t*_3_).

The following hypotheses are to be tested:

- H1: Students’ perceived P-E-F in sixth grade indirectly and negatively predicts them choosing a non-qualifying upper secondary education at the end of ninth grade.
- H2: This effect is mediated by self-concepts in mathematics and expectations in mathematics (H2a) and by self-concepts in German and expectations in German (H2b) in lower secondary school.
- H3: Students’ self-concept in mathematics in seventh grade has a negative indirect effect on them choosing a non-qualifying upper secondary education at the end of ninth grade, conveyed via expectations in mathematics in ninth grade.
- H4: Students’ self-concept in German in seventh grade has a negative indirect effect on them choosing a non-qualifying upper secondary education at the end of ninth grade, conveyed via expectations in German in ninth grade.

**Fig. 1:**
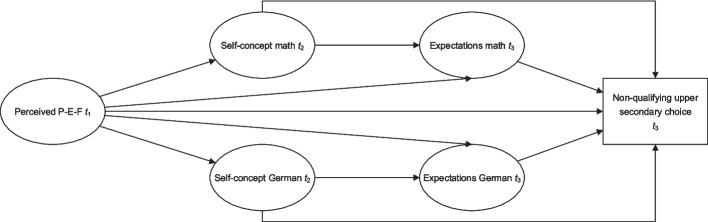
Working model depicting the tested paths between perceived P-E-F, self-concepts, expectations of success, and a non-qualifying upper secondary education

The assumed model is depicted in Fig. 1. Direct paths from perceived P-E-F on expectations in mathematics and German as well as on upper secondary education are included to test whether the mediation is full or partial. Since upper secondary educational choices also depend on the school level visited at lower secondary school (Meyer & Scacchi 2020), it will be controlled for in the analysis.

In line with statistical convention, we use the terms “predict” and “effect” to describe modeled regression paths. These terms refer to statistical prediction and estimated path coefficients, not to causal effects (Cohen et al., 2003). Given the observational design, all coefficients are interpreted as associations rather than causal influences.

## Method

### Participants and procedure

The hypotheses were tested using data from the Swiss longitudinal study WiSel ("Wirkungen der Selektion"; "Effects of Tracking"), in which data was collected from students from various Swiss cantons (Argovia, Basel Country, Berne, Lucerne) in six survey waves. Schools were randomly selected within these cantons. For the present study, data from survey waves 2 (*t*_1_; school year 2012/2013; sixth grade), 3 (*t*_2_; school year 2013/2014; seventh grade), and 4 (*t*_3_; school year 2015/2016; ninth grade) were used. At all three measurement points, data was collected using a questionnaire that the students completed during lesson time.

For the analysis, all participants for whom information was available as to (a) which school level they were assigned to in lower secondary school and (b) which upper secondary education they would begin were initially selected (*N* = 645). This sample was then specified further, and only those students who lived in cantons where the transition to lower secondary school took place between *t*_1_ and *t*_2_ (Berne and Lucerne) were included. This ensured that all students experienced the transition at the same time, allowing for a consistent longitudinal analysis and resulted in a final sample of *N* = 388 adolescents (48.2% = female, 51.8% = male; *M*_Age_ at *t*_1_ = 11.8).

Of these 388 adolescents, 174 (44.8%) participated at all three measurement points, while 214 (55.2%) participated at two (*t*_2_ and *t*_3_). To test for missing response patterns, *t*-tests were conducted in SPSS 28 between these two groups, for all *t*_3_ items. There were no significant differences concerning any of the items, indicating no group differences. All 388 participants were included in the final structural equation model.

### Measurements

Adolescents’ upper secondary education was assessed at the end of the ninth school year (*t*_3_). Teachers were asked to indicate what upper secondary education each of their students was going to begin in the following school year. This information was therefore collected a few months after the other *t*_3_ variables. A binary variable was created, with all adolescents who would start a qualifying upper secondary education (i.e., general education and VET) in one group and all adolescents who had not found a qualifying education (i.e., interim solution or no upper secondary education at all) in another.

Expectations of success in mathematics and German were assessed during the ninth school year (*t*_3_), using the same three items for each subject, translated from Wigfield and Eccles (2000) as well as the Michigan Study of Adolescent and Adult Life Transition (MSALT, n.d.). The items focused on adolescents’ expectations concerning their success at performing in the respective areas (e.g., “How well would you do in a job that requires good mathematics/German skills?”). Participants answered on a Likert scale ranging from 1 (“not well at all”) to 6 (“very well”). Both scales showed good reliability (mathematics: *α* =.82; German: *α* =.85).

Self-concepts in mathematics and German were assessed in seventh grade (*t*_2_) with four items per subject, modified after a scale by Neuenschwander et al. (2007). Unlike the items on expectations of success, these scales did not focus on future performance, but rather on the current subjective assessment of one’s skills (e.g., “Participating in mathematics/German class is easy for me”). Responses ranged from 1 (“not true at all”) to 6 (“totally true”) on a Likert scale. Reliability was good for both scales (mathematics: *α* =.89; German: *α* =.91).

Perceived person–environment fit was assessed in sixth grade (*t*_1_), using five items by Neuenschwander et al. (2009). The items encompassed different aspects of perceived person–environment fit: fit to one’s abilities (e.g., “I can use my strengths in class”), fit to one’s emotional needs (e.g., “I feel comfortable in class”), and fit to one’s interests (e.g., “I get interesting tasks in class”). The reliability of the scale was good (*α* =.81).

The *school level* into which the students were divided was surveyed at *t*_2_, after they entered lower secondary school. For the present study, a binary variable was created in which all students attending the lowest school level were divided into one group and those at the intermediate or high level into the other.

### Data analysis

To test the hypotheses, a structural equation model (SEM) was estimated, using Mplus 8.6 (Muthén & Muthén, 1998–2017). Since individual cases were clustered within school classes, we used the TYPE = COMPLEX function. This approach adjusts for the nested data structure and accounts for potential dependencies within clusters, ensuring more accurate standard errors and parameter estimates (Muthén & Muthén, 1998–2017).

The evaluation of model adequacy was based on the *χ*^2^ statistics, comparative fit index (CFI), root mean square error of approximation (RMSEA), and standardized root mean square residual (SRMR; Kline, 2005; Schermelleh–Engel et al., 2003).

Before calculating the SEM, an MCAR test (Missing Completely at Random; Little, 1988) was performed in SPSS 28 for all included items. The MCAR did not provide significant results, which means that all missing values were completely at random. Since the necessary requirements were met, missing values were processed using maximum likelihood (ML) estimation (Boomsma, 2000), which is a widely accepted method in structural equation modeling, particularly when data are missing at random (MAR) or completely at random (MCAR; Olinsky et al., 2003; Shin et al., 2022). ML estimation has been shown to produce efficient and unbiased parameter estimates under these conditions and, in SEM contexts, is often preferable to multiple imputation (Allison, 2012; Enders, 2010).

In addition to the initial SEM, a secondary model was developed to examine the robustness of the results by additionally including grades in mathematics and German as control variables. This model will be presented in the Appendix.

Since the hypotheses are directed, one-sided* p* values are reported.

## Results

### Descriptive results and correlations

Descriptive statistics and bivariate correlations are depicted in Table 1. Grades in mathematics and German, which are part of the secondary model depicted in the Appendix, are included. Notably, a non-qualifying upper secondary education did not significantly correlate with expectations of success in German, suggesting that this assumed direct effect does not exist.

**Table 1:** Means and standard deviations of latent variables and Pearson correlations among all variables

	*N*	*M*	*SD*	1	2	3	4	5	6	7	8	9
1 Perceived P-E-F *t*_1_	173	4.79	0.62	-								
2 Self-concept mathematics *t*_2_	385	4.43	0.83	.13*	-							
3 Self-concept German *t*_2_	385	4.54	0.75	.34***	.07	-						
4 Expectations mathematics *t*_3_	388	4.21	1.03	.12*	.52***	.01	-					
5 Expectations German *t*_3_	386	4.41	0.83	.24**	−.09*	.44***	−.06	-				
6 Non-qualifying upper secondary education *t*_3_	388	-	-	−.11	−.13**	−.13**	−.18***	.01	-			
7 Lower secondary school level *t*_2_	388	-	-	−.02	−.09*	.08	−.04	.08	.18***	-		
8 Grade mathematics *t*_3_	380	5.54	1.30	.08	.36***	.03	.39***	-.08	−.10*	.01	-	
9 Grade German *t*_3_	381	5.58	0.95	.13*	.05	.24***	.09*	.37***	−.13**	−.12**	.22***	-

*p <.05; **p <.01; ***p <.001

## Test of hypotheses

**Fig. 2:**
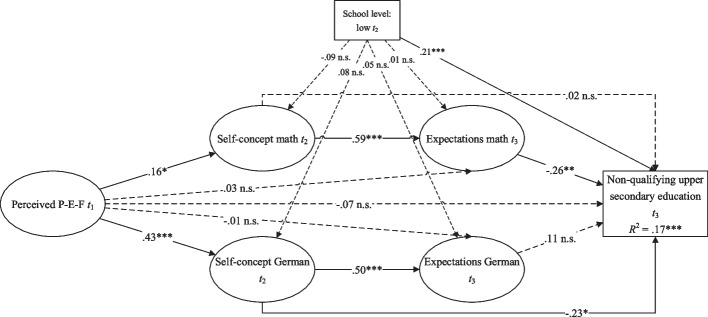
Structural equation model illustrating the direct and indirect effects of perceived P-E-F, self concepts, and expectations of success on a non-qualifying upper secondary education. Notes: *p ≤.05; **p ≤.01; ***p ≤.001; R2: Explained variance tTest of robustness

The final SEM (Fig. 2) showed acceptable fit indices (*χ*^2^ (175) = 250.2, *χ*^2^/*df* = 1.43,* p* <.001, CFI =.94, RMSEA =.03, SRMR =.06). Students’ perceived P-E-F at *t*_1_ positively predicted their self-concept at *t*_2_ in mathematics (*β* =.16,* p* =.035) as well as in German (*β* =.43,* p* <.001). Both self-concepts positively predicted expectations of success in the respective subject at *t*_3_ (mathematics: *β* =.59,* p* <.001; German: *β* =.50,* p* <.001). A non-qualifying upper secondary education at *t*_3_ was negatively predicted by expectations of success in mathematics (*β* = –.26,* p* =.002), but not German (*β* =.11,* p* =.138). Notably, however, there is a direct negative effect of the self-concept in German at *t*_2_ (*β* = –.23,* p* =.026).

Perceived P-E-F at *t*_1_ indirectly and negatively predicted a non-qualifying upper secondary education at *t*_3_ (*β* =.10,* p* =.024; H1 supported) via self-concept at *t*_2_ and expectations of success at *t*_3_ in mathematics (*β* = −.02,* p* =.05; H2a supported), but not in German (*β* =.02,* p* =.135; H2b rejected). There was, however, an indirect effect via self-concept in German (*β* = −.10,* p* =.035).

Students’ self-concept at *t*_2_ had an indirect negative effect on a non-qualifying upper secondary education at *t*_3_ via expectations of success at *t*_3_ in mathematics (*β* = −.15,* p* =.002; H3 supported), but not in German (*β* =.06,* p* =.139; H4 rejected).

The secondary model that included grades as control variables confirmed the findings of the primary model. The findings of this secondary model can be found in the Appendix.

## Discussion

The aim of the present study was to analyze whether students’ perceived P-E-F in primary school could indirectly predict whether they would enter a non-qualifying upper secondary education at the end of ninth grade, mediated by self-concepts and expectations of success in mathematics and German. The results are consistent with this assumption, with the present study supporting the mediating role of motivational beliefs across different school years and academic domains. Specifically, perceived P-E-F in sixth grade predicted self-concepts in both mathematics and German in seventh grade, which in turn predicted the respective expectations in ninth grade. This finding aligns with previous studies that found effects between students’ P-E-F, self-concepts, and expectations of success (e.g., Dickhäuser & Reinhard, 2006; Midkiff et al., 1986; Urdan & Argueta-Vogel, 2018). The present study shows that these associations are furthermore indirectly related to later educational outcomes, such as whether students enter a non-qualifying upper secondary track.

A key finding is the difference in how self-concepts and expectations of success relate to adolescents’ educational trajectories between mathematics and German. In mathematics, expectations of success fully mediated the effect of self-concept on the choice of a non-qualifying upper secondary education, whereas in German, self-concept had a direct effect and expectations of success did not significantly contribute. This indicates that domain-specific mechanisms underlie the relationship between self-concept, expectations of success, and educational choices. A possible explanation for this could be that a lot of job opportunities in VET demand mathematical skills. Therefore, expectations of success in mathematics may directly predict adolescents’ decisions to pursue a qualifying upper secondary education. In contrast, the self-concept in German may reflect more general self-esteem in communicative situations (social situations, such as job interviews). These situations play a key role in securing an apprenticeship (Imdorf, 2007), while specific task-related skills (reading and writing proficiency) are less predictive of success in certain upper secondary education paths. Previous research has also shown that students’ self-concept in German tends to be less differentiated, meaning that it is more broadly linked to general self-perceptions such as general academic confidence and verbal ability and therefore more likely to influence other areas beyond just academic performance in the subject (Schuchardt et al., 2015). This may also explain why self-concept in German has a direct effect on the choice of the upper secondary education: language proficiency is relevant across multiple domains and may therefore reflect a broader sense of confidence. Person-related beliefs, such as self-concepts, may play a more prominent role in German, while task-related beliefs, such as expectations of success, could be more crucial in mathematics. This observation adds nuance to the assumptions of expectancy-value theory, which posits an effect of self-concepts on expectations of success (Eccles & Wigfield, 2002) but does not fully address domain-specific variations. Further research should explore how person- and task-related beliefs affect educational choices across different domains and contexts.

The present study’s findings highlight the importance of perceived P-E-F in primary school and its longitudinal effects on student’s motivational beliefs and subsequent educational outcomes. The significant indirect effect on a non-qualifying upper secondary education underscores the role of a fitting primary school environment, as it is not only associated with students’ motivational beliefs but also their educational trajectories in the long term. These results align with previous research (e.g., Gerber-Schenk etal., 2010) that suggests that a matching learning environment enhances self-concepts. Notably, this seems to be a longitudinal effect, persisting over three school years, including the transition to lower secondary school.

By focusing on the longitudinal associations of perceived P-E-F, this research may offer tentative implications for educational practice. If the findings can be replicated and future research confirms causal relations, they would suggest that educators in primary school may be able to foster conditions that are associated with students’ later educational success by helping them experience a stronger sense of P-E-F. Ways to do this include nurturing positive teacher-student relationships (Hagenauer & Raufelder, 2021; Ramseier & Neuenschwander, 2024a; Zimmer-Gembeck et al., 2006), maintaining a positive class climate and classroom community (Ramseier & Neuenschwander, 2024b), and ensuring tasks are appropriately challenging for students’ abilities (Eccles et al., 1993). Furthermore, students’ self-concept and expectations of success also appear to be associated with a successful transition to a qualifying upper secondary education. Further research should examine whether strengthening these beliefs during lower secondary school—for example, through constructive feedback and self-referenced orientation when grading (e.g., Rheinberg, 2006)—can contribute to improved educational trajectories.

The present study is consistent with expectancy-value theory by additionally considering P-E-F, offering a broader perspective of how motivational beliefs are associated with educational outcomes. By combining these two approaches, the present study indicates that students’ perceptions of their learning environment, in the shape of P-E-F, are linked to their self-concepts and expectations of success in subsequent school years and, consequently, of their educational success. Further studies are needed to test whether these associations reflect causal processes.

## Limitations

The present study is subject to certain limitations. The constructs were recorded exclusively on the basis of the participants’ perceptions. It is possible that an objective measurement of person-environment fit would yield different results, though perceived P-E-F has been shown to be a reliable predictor of educational choices (Nägele et al., 2017).

Additionally, self-concepts and expectations of success were only measured at one point each, which prevents us from making a statement about the development of these constructs over time or possible reciprocal effects. Including repeated measures of self-concepts and expectations in future models would allow for a more nuanced understanding of how these motivational beliefs change across different educational stages.

While the sample was drawn from two cantons (Berne and Lucerne), these regions were chosen to ensure participants had a consistent transition timing to lower secondary school. Future studies could confirm these findings across additional regions or educational contexts.

An additional consideration concerns the absence of a significant total effect (*β* = −0.18, *p* =.078) of perceived P-E-F on students’ educational choices. It is possible that other unmeasured variables (e.g., perceived academic pressure, social influences) may have opposing effects on educational choices. Future research could explore whether such variables help explain the overall pattern.

A further limitation concerns different levels of abstraction between the measured constructs: perceived P-E-F is broad whereas self-concepts are domain-specific. Future work should further differentiate these constructs.

Lastly, while the study’s longitudinal design allows us to examine the temporal sequence of relationships between the constructs, it is important to note that the study is observational in nature. As such, causality cannot be inferred. Although we hypothesized that perceived P-E-F in sixth grade indirectly predicts the educational choice in ninth grade through its effects on self-concepts and expectations of success, the assumed temporal direction of these effects cannot be definitely confirmed. Our theoretical framework (Eccles & Wigfield, 2002; Eccles et al., 1993) suggests that students’ perceptions of their learning environment relate to their motivational beliefs, which in turn are associated with their subsequent educational decisions. However, it remains possible that the relationship between perceived P-E-F and self-concepts is bidirectional, with prior self-concepts influencing students’ perceived P-E-F. Future research employing experimental or cross-lagged designs could help further clarify causal pathways between these constructs.

## Conclusion

In summary, the present study highlights the potential relevance of perceived P-E-F in relation to students’ academic self-concepts and expectations of success, which, in turn, are associated with adolescents entering a non-qualifying upper secondary education. Our findings suggest perceived P-E-F in primary school may represent a valuable focus in educational practice, particularly if future research confirms its association with motivational beliefs and educational trajectories. Creating a learning environment that aligns with students’ abilities, needs, and interests may help foster more positive academic self-concepts and expectations, which are associated with more favorable educational transitions.

While the results point to possible links between perceived P-E-F and domain-specific motivational beliefs, future research should continue to explore the domain-specific nature of motivational beliefs and how they relate to educational choices across various subjects and educational systems. Understanding these mechanisms more deeply can inform targeted interventions that help reduce the risk of students entering non-qualifying educational paths.

## Figures and Tables

**Fig. 3:**
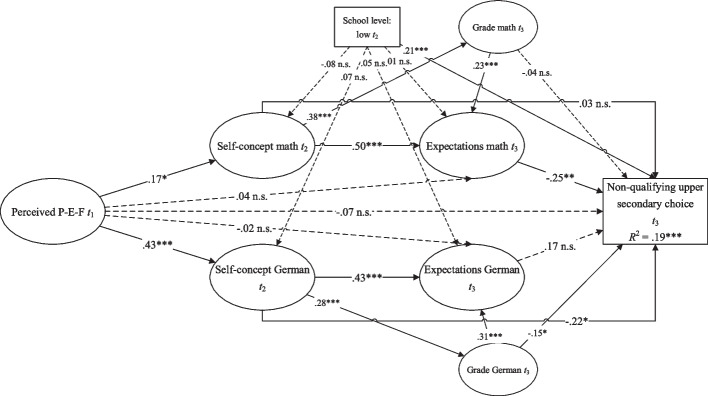
Alternative structural equation model illustrating the direct and indirect effects of perceived P-E-F, self-concepts, and expectations of success on a non-qualifying upper secondary education, including grades. *Notes*: *p ≤.05; **p ≤.01; ***p ≤.001; *R*^2^: explained variance

## Data Availability

The data supporting the findings of this study are archived in the SwissUbase repository (https://www.swissubase.ch) and are available under restricted access conditions. Reference numbers: 11063 and 12206.

## Appendix

Detailed test of robustness

To test the robustness of the results, an additional SEM with grades in mathematics and German in eigh8th grade was calculated. Since the present study uses the framework of expectancy-value theory, which does not include effects of grades on educational choices (Eccles & Wigfield, 2002), the model without grades was used for the main analysis. However, as students’ grades contribute to their decision to an upper secondary education track in Switzerland (Neuenschwander, 2012), an additional model will be presented here. The grades were surveyed in nin9th grade (t3), when students were asked to provide their last grades in German and mathematics, i.e., the final grades of the eigh8th grade. These are the grades that are generally most important for finding an upper secondary education, since adolescents look for an education during nin9th grade and usually apply with their eigh8th grade certificate. In Switzerland, grades range from 1 to 6, with 6 being the best possible grade. Grades below 4 are seen as insufficient (failed). This second model’s fit was initially problematic, due to a low CFI (χ2 (211) = 439.204, χ2/df = 2.08, *p* <.001, CFI =.81, RMSEA =.05, SRMR =.03). Modification indices showed that a path from self-concepts in mathematics and German on the grades in the respective subject needed to be included to improve the model’s fit. This additional path is not part of the theoretical framework, but can be justified, due to the known connection between self-concepts and achievement in the respective domain (Marsh & Martin, 2011). The adjusted model (Fig. 3) showed acceptable fit indices (χ2 (212) = 302.672, χ2/df = 1.43, *p* <.001, CFI =.93, RMSEA =.03, SRMR =.06). Students’ perceived P-E-F at t1 positively predicted their self-concept at t2 in mathematics (β =.17, *p* =.029) as well as in German (β =.43, *p* <.001). Both self-concepts positively predicted expectations of success in the respective subject at t3 (mathematics: β =.50, *p* <.001; German: β =.43, *p* <.001). A non-qualifying upper secondary education at t3 was negatively predicted by expectations of success in mathematics (β = –.25,-.25, *p* =.005), but not German (β =.17, p =.072). Perceived P-E-F at t1 indirectly and negatively predicted a non-qualifying upper secondary education at t3 (β =.06, *p* =.026). Students’ self-concept in mathematics at t2 had an indirect negative effect on a non-qualifying upper secondary education at t3 via expectations of success at t3 (β = –.12,-.12, *p* =.005);.005;), but not in German (β =.07,* p* =.071). The model with grades in mathematics and German as control variables therefore confirms the results of the primary model.
